# Using bio.tools to generate and annotate workbench tool descriptions

**DOI:** 10.12688/f1000research.12974.1

**Published:** 2017-11-30

**Authors:** Kenzo-Hugo Hillion, Ivan Kuzmin, Anton Khodak, Eric Rasche, Michael Crusoe, Hedi Peterson, Jon Ison, Hervé Ménager

**Affiliations:** 1 Bioinformatics and Biostatistics HUB, Centre de Bioinformatique, Biostatistique et Biologie Intégrative (C3BI, USR 3756 Institut Pasteur et CNRS), Paris, France; 2 Institute of Computer Science, University of Tartu, Tartu, Estonia; 3 Igor Sikorsky Kyiv Polytechnic Institute, National Technical University of Ukraine, Kyiv, Ukraine; 4 Lehrstuhl für Bioinformatik, Institut für Informatik, Albert-Ludwigs-Universität Freiburg, Freiburg, Germany; 5 Common Workflow Language Project, Vilnius, Lithuania; 6 DTU Bioinformatics, Technical University of Denmark, Copenhagen, Denmark

**Keywords:** bioinformatics, tool integration, galaxy, common workflow language, interoperability, registry

## Abstract

Workbench and workflow systems such as Galaxy, Taverna, Chipster, or Common Workflow Language (CWL)-based frameworks, facilitate the access to bioinformatics tools in a user-friendly, scalable and reproducible way. Still, the integration of tools in such environments remains a cumbersome, time consuming and error-prone process. A major consequence is the incomplete or outdated description of tools that are often missing important information, including parameters and metadata such as publication or links to documentation. ToolDog (Tool DescriptiOn Generator) facilitates the integration of tools - which have been registered in the ELIXIR tools registry (https://bio.tools) - into workbench environments by generating tool description templates. ToolDog includes two modules. The first module analyses the source code of the bioinformatics software with language-specific plugins, and generates a skeleton for a Galaxy XML or CWL tool description. The second module is dedicated to the enrichment of the generated tool description, using metadata provided by bio.tools. This last module can also be used on its own to complete or correct existing tool descriptions with missing metadata.

## Introduction

Over the last few years, bioinformatics has played a major role in the field of biology, raising the issue of best practices in software development for the members of the bioinformatics community ^1– 3^. These practices include facilitating the discovery, deployment, and usage of tools, and several helpful solutions are available.

Tool discovery is facilitated by various online catalogs and registries ^4– 6^. The ELIXIR Tools and Data Services Registry, bio.tools ^7^, describes bioinformatics software using extensive metadata descriptions, supported by the EDAM ontology ^8^.

For software deployment, distribution systems are available ^9– 13^ that let users locally install the tools that they need in convenient, portable and reproducible ways. Workbench and workflow systems such as Galaxy ^14, 15^, Taverna ^16^ or Chipster ^17^ allow the execution and composition of bioinformatics tools in integrated environments which aim at improved usability, interoperability and reproducibility. Finally, the Common Workflow Language ^18^ (CWL) is a recent project that defines a standardized and portable tool and workflow description format, usable across different platforms.

**Figure 1.**
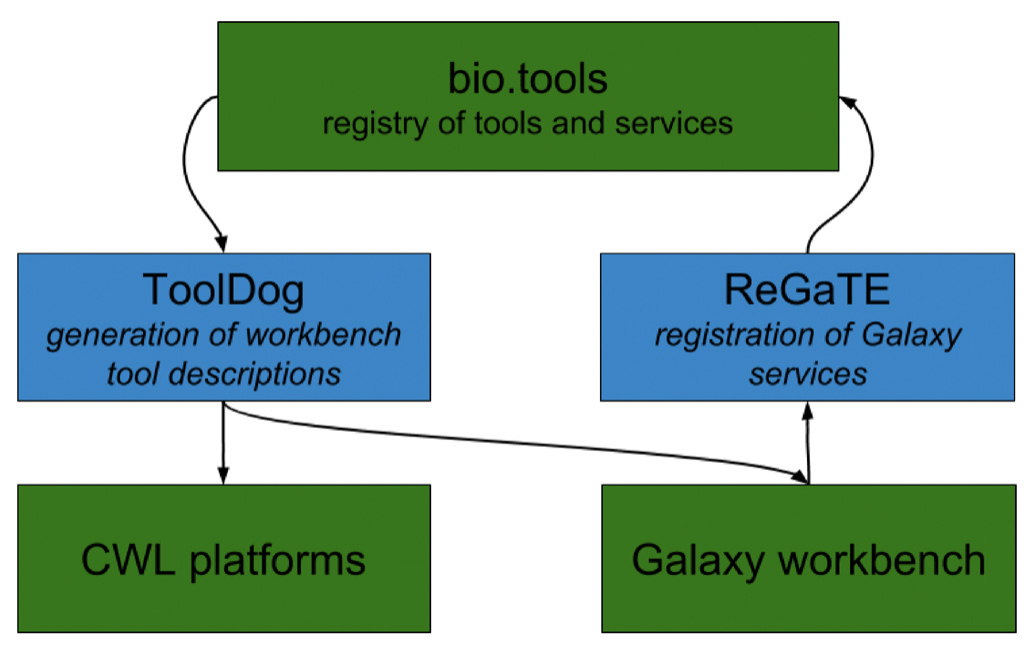
Workbench Integration Enabler overview. The objective is to integrate the bio.tools registry with workbench environments in two ways: (1) “ReGaTE”, a utility for *en masse* registration of services from Galaxy instances; (2) the “ToolDog” utility, to translate the description of any tool or service that is registered in bio.tools, into the format required by the existing major workbench environments.

All of the above systems rely on components that provide the necessary information to describe, install, or run a specific piece of software. Gathering this information and formatting it into tractable tool descriptions is often a complex and time consuming task for developers. Indeed, it requires a deep knowledge of both the tool itself and the description format. A significant part of the metadata stored in the descriptions is, however, common to registries and workbench environments systems ^19^, and strategies relying on a mapping between these different description formats can help avoid redundancy and mislabeling of tools Figure 1). The ReGaTE utility ^20^ illustrates this by using tool descriptions from Galaxy to publish available services on bio.tools. Another application is to facilitate workbench environment integration, by reusing tool descriptions from registries. Here we present “ToolDog” (Tool DescriptiOn Generator), an application that enables workbench integration for tools registered in the bio.tools registry.

### Tool descriptions

Bioinformatics tools are described in various formats and levels of detail, befitting different systems and use-cases. A bio.tools entry provides tool descriptions for tool end-users, primarily for search and discovery purposes. The metadata provides a basic description including the tool type, what task it performs, the main input and output data, who created it, where it is available, and its license. This description, based on the BiotoolsSchema model, can be accessed through the bio.tools API and retrieved in JSON format. Conversely, Galaxy and CWL tool descriptions must support tool discovery, execution, and integration into homogeneous environments. This requires an extensive description of their command line syntax (or other type of API). Galaxy tool descriptions are written in XML or YAML, and the corresponding XSD is available. CWL tool descriptions are described using the YAML-based SALAD format.

All three of these tool description formats provide the possibility of specifying EDAM terms. In bio.tools this can be done directly. CWL supports these annotations through the addition of bioschemas mark-up, and Galaxy supports EDAM through specific tags mapping to its internal typing system ^21^. The EDAM ontology helps with the description of the tools by providing a common vocabulary that includes terms to describe topics that specify which particular domains of bioinformatics the tool serves, operations that describe what the tool does, and data and formats that specify the type and format of the inputs and outputs.

### Completeness of Workbench tool description

**Figure 2.**
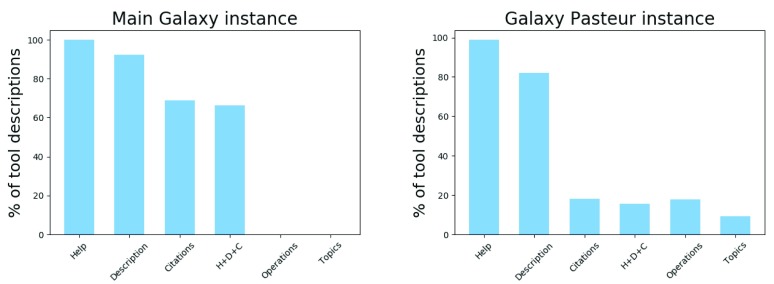
Metadata coverage for Galaxy tool descriptions from ( **A**) the main Galaxy instance ( https://usegalaxy.org) and ( **B**) the Institut Pasteur Galaxy instance ( https://galaxy.pasteur.fr). The graphs show the percentage of tools possessing various metadata types: *Help*: usage instructions; *Description*: description of the tool to be displayed in the tool menu; *Citations*: tool citation information using either a DOI or a BibTeX entry; *H+D+C*: contains a help, description and citations section; *Operations*: description of the EDAM operation(s) performed; *Topics*: description of the EDAM topics covered. The total number of tools includes those which were successfully retrieved and analyzed (672 out of 1209 on Galaxy main, 351 out of 526 on Pasteur); not all available tools were retrieved - some because they are not available in a ToolShed, and some because we chose to retrieve only the latest version of each tool and discarded the earlier ones.

Tool descriptions for workbench systems are expensive to create and maintain, because they require exhaustive knowledge of both the described tool, and the syntax used for the description ^19^. Consequently, tool descriptions are sometimes incomplete or out of date. For instance, in the case of Galaxy, the analysis of the main server and the server of the Institut Pasteur ^22^ shows that some tools are not adequately described (see Figure 2). Specifically, although most of the tools have a help section and a description, important elements such as citation information are often missing. The evolution of the Galaxy framework itself also generates a need for maintenance, through changes in the tool description format. With the recent addition of EDAM annotations tags in the format, tools had to be updated to support this new feature. The users of such graphical workbench platforms do not typically handle tool discovery and deployment tasks. Thus, detailed tool descriptions are fundamental, because they are the main source of information for the scientists who use them.

Different approaches exist to help improve the quality of the corpus of tool descriptions. (1) Tooling facilitates the creation and validation of the tool descriptions, using Planemo ^23^ in the case of Galaxy. (2) Community approaches such as the Intergalactic Utilities Commission design and promote best practices for the development of Galaxy tools. (3) Standardization efforts like CWL also reduce the maintenance work for tool descriptions by making them portable between different platforms.

ToolDog complements all of these approaches. It leverages the information available in bio.tools to simplify the integration of bioinformatics software into workbench environments.

## Methods

**Figure 3.**
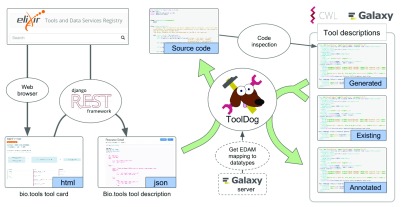
ToolDog generates tool descriptors from bio.tools resources descriptions.

ToolDog is a command-line utility written in Python. It consists of two modules, which handle (1) the generation of a skeleton for the tool description, based on the analysis of the source code of the tool, and (2) the enrichment of the tool description, using the bio.tools metadata. The tool description generation pipeline ( Figure 3) leverages bio.tools and includes both a module to generate a tool description using only the registry, as well as a module to enrich an existing tool description with information from the registry.

### Source code analysis

For a number of bioinformatics tools, a significant part of their description can be extracted from an analysis of the source code. The source code analysis module of ToolDog does this, currently only with python-based tools that use the *argparse* library for parsing command-line arguments. This module uses the argparse2tool package to retrieve the list of parameters and generate Galaxy or CWL tool description skeletons. To generate such skeletons, ToolDog runs a Docker software container that will download, install, analyze the source code, generate the tool description and then retrieve it. This strategy avoids the pollution of the local user’s environment and provides a completely pre-configured, ready-to-use installation of ToolDog.

### Tool description enrichment

Galaxy and CWL tool descriptions, whether they were manually authored or automatically generated by source code analyses, can be improved by the description enrichment module. This retrieves additional metadata from the corresponding bio.tools entries, and fills in the missing information in the workbench tool description when available.

Internally, the input tool description is parsed into an object model of the tool. The metadata from bio.tools are then mapped onto this object model, which is later exported to Galaxy or CWL formats. Parsing and export capabilities of ToolDog leverage the galaxyxml or cwlgen libraries to import and export the updated descriptions.

## Results

### Generation of a tool description from a bio.tools entry

**Figure 4.**
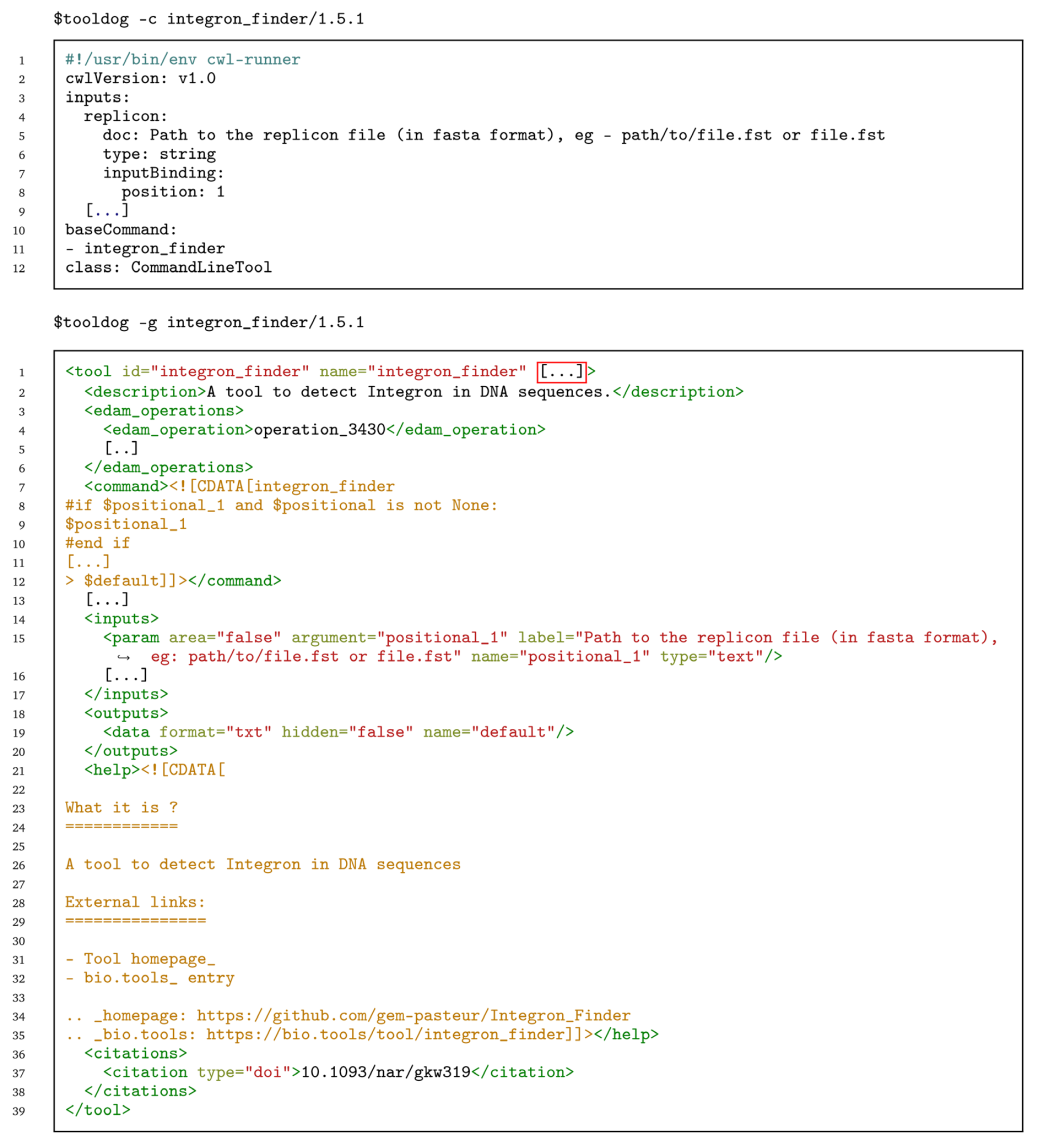
Output of the run of ToolDog using the bio.tools entry of IntegronFinder to generate the corresponding CWL and Galaxy tool descriptions.

Here we illustrate the generation of a tool description with the example of IntegronFinder ^24^, an analysis tool dedicated to the identification of integrons in bacterial genomes. Launching ToolDog in “generation mode” on the IntegronFinder entry in the bio.tools registry allows the generation of a significant portion of the tool description ( Figure 4), either in CWL or Galaxy format. Some manual modifications (corrections + additions) are still necessary to complete the tool description and to make it functional. For instance, software requirements, which specify what software needs to be installed for the tool to run correctly, cannot be automatically generated, because this information is currently not available in bio.tools. Additionally, the mapping between inputs and the generated command line, as well as between outputs and the file names they refer to is not present.

### Enrichment of an existing collection of tool descriptions

**Figure 5.**
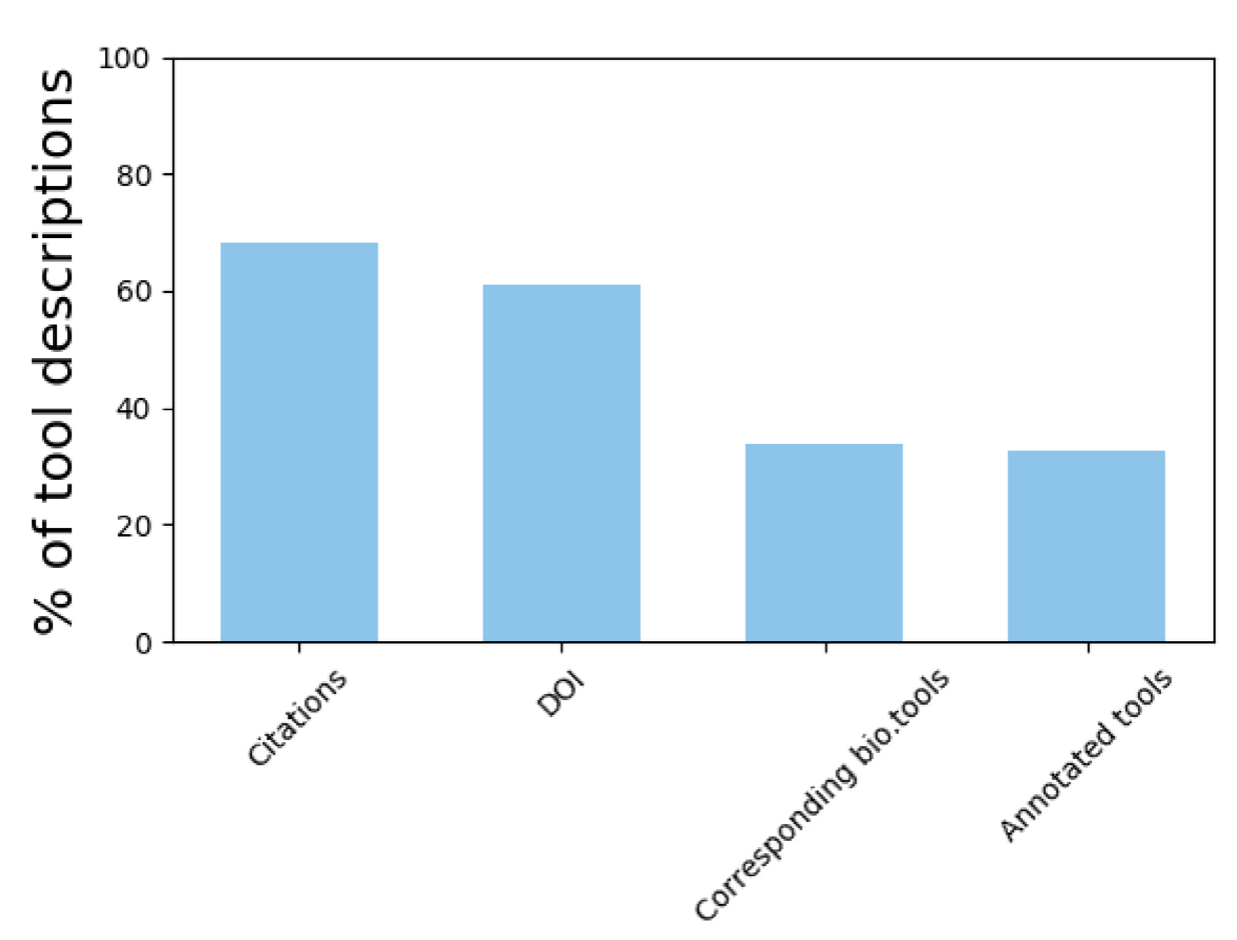
Tool descriptions automated mapping and enrichment. Out of 665 retrieved tool descriptions, 399 have a DOI and 224 of these descriptions could be mapped to a bio.tools entry. 217 tool descriptions have been successfully annotated using ToolDog ( *Citations*: presence of tool citation information; *DOI*: tool citation information described using a DOI; *Corresponding bio.tools*: tool descriptions with a corresponding bio.tools entry retrieved using the DOI; *Annotated tools*: tool descriptions successfully annotated with ToolDog).

In addition to novel tool description generation, ToolDog can also perform the automated enrichment of existing tool descriptions with bio.tools metadata. To test this approach, we ran ToolDog on the tool descriptions available on the Galaxy main instance that lack EDAM annotations. All of the Galaxy descriptions from the main instance were retrieved, and mapped to bio.tools entries using the citation identifiers (DOI). The goal was to add EDAM terms describing the topic of application and the operation(s) performed by the tools. To avoid linking unrelated entries, we took a conservative approach, only mapping by default two entries when they referred to, and only to, the same publication. The results ( Figure 5) show that whenever this linking can be reliably done, the enrichment can easily be performed, with a total of 217 Galaxy tool descriptions being enriched out of 224 being initially mapped to bio.tools. A detailed description of this analysis, including the original and annotated tool descriptions, is available at https://github.com/khillion/galaxyxml-analysis/annotate_usegalaxy.

## Discussion

The ToolDog utility allows a developer to generate new tool descriptions for tools which are compatible with the code analysis module, and reuse the metadata provided by bio.tools to enrich existing tool descriptions. There are some limitations to this approach:

1. The “plugin” libraries used for code analysis are specific to the programming languages, libraries or framework used to build the command line interface. To this date, they don’t cover most of these.
2. The generation of the tool descriptions through code analysis must assume certain coding practices, such as the use of specific functions to define input or output parameters, which are not uniformly adopted.
3. Some of the input/output operations performed by some programs are a lot more difficult to detect through code analysis because they are typically not included in command line parsing frameworks, such web service and database queries and submissions, or in place file modifications.

The automated enrichment of existing tool descriptions provides a convenient way to improve them, especially if they lack most of the metadata provided by bio.tools. Performing this enrichment efficiently *en masse*, however, would require the wide adoption of an identification system for bioinformatics software. This mechanism would allow to avoid the complex and sometimes ambiguous mapping procedures based on publication identifiers we performed when testing it on the Galaxy tools. A recent update to bio.tools has added stable and unique tool identifiers, based on registered tool names, yielding persistent references to tools, for example https://bio.tools/signalp. Future work will make use of these identifiers to improve the generation of tool descriptions. For instance, linking of the bioconda and biocontainers repositories to bio.tools will enable ToolDog to generate software requirements compatible with workbench platforms ^25^.

## Conclusions

During the last years, integration of various tools has been eased by the use of workbench systems such as Galaxy, and frameworks using the Common Workflow Language. Still, it remains time consuming and not straightforward to adapt resources to such environments. ToolDog lays the foundation for future work, that will provide a Workbench Integration Enabler for the bio.tools registry as an online service. Furthermore, integration with Planemo, the main utility to develop Galaxy and CWL tools, will be further developed in order to make the simple, bio.tools-based metadata enrichment of ToolDog available to the widest possible audience.

## Data availability

The scripts and results of the analysis performed to motivate and test our approach are available at: https://github.com/khillion/galaxyxml-analysis, and are archived at the time of publication at: https://doi.org/10.5281/zenodo.1038005 ^26^.

## Software availability

The ToolDog software is available at: https://pypi.python.org/pypi/tooldog

The source code is available at: https://github.com/bio-tools/tooldog

Archived source code as at the time of publication: https://doi.org/10.5281/zenodo.1037909 ^27^

Software license: MIT License.
