# Supplementary material for: Using bio.tools to generate and annotate workbench tool descriptions
Source: F1000Res. 2017 Nov 30;6:ELIXIR-2074. [Version 1] doi: 10.12688/f1000research.12974.1 (PMC5747335; doi:10.12688/f1000research.12974.1)
Supplement: Peer review history

# Peer review: Referee response for version 1

**DOI:** 10.5256/f1000research.14069.r28595

**Published:** 2018-01-16

**Authors:** Brian O'Connor

**Affiliations:** 1 Computational Genomics Platform, UCSC Genomics Institute, University of California, Santa Cruz, Santa Cruz, CA, USA

"Using bio.tools to generate and annotate workbench tool descriptions" is an article that describes a tool descriptor program known as ToolDog. It was designed to generate Galaxy XML or CWL from particular bioinformatics tool source code as well as metadata annotations on bio.tools. The idea is great, since the issue is a real one in the community. Namely, there are a lot of tools out there but typically they lack descriptors in Galaxy of CWL format. And this makes it harder to use in "workbench" and workflow systems. Creating a tool that tool authors can use to help create descriptors is awesome. Source is available in GitHub and the tool can be installed via pip.  

 Feedback/Questions

1. Can the authors rename the article? I think it should include ToolDog in the article title.
2. What are the plans for other languages (if any)? Do the authors see ToolDog as something that others will extend for, say, WDL generation?
3. I think it would be interesting to hear more about future plans. Specifically, how will the authors expand this to a Workbench Integration Enabler? Do they see this as being an automated process? How will they leverage the work of bioconda and biocontainers (they did mention this briefly) and will the goal be to generate CWL/GalaxyXML for everything in bio.tools + bioconda/biocontainers?  
4. Alternatively, if the goal not to automatically export CWL/GalaxyXML for everything in bio.tools, is it, instead, to provide a tool for tool authors to use when building their tool to jumpstart their descriptor creation? Some clarification on the intended audience I think would be helpful.
5. The authors described generating CWL/Galaxy XML for IntegronFinder. Did they try other tools and, if so, how successful was that? What about generation in bulk?
6. Can they comment on what a tool author should do with the generated CWL or Galaxy XML? They mention in the results that some work is required to make the tool run correctly. Is the tool author then suggested to check in the CWL/Galaxy XML to their source repo and maintain it? What is the recommendation here?

I have read this submission. I believe that I have an appropriate level of expertise to confirm that it is of an acceptable scientific standard.

# Peer review: Referee response for version 1

**DOI:** 10.5256/f1000research.14069.r28567

**Published:** 2018-01-10

**Authors:** Manuel Corpas

**Affiliations:** 1 Cambridge Precision Medicine, Cambridge, UK

The article 'Using bio.tools to generate and annotate workbench tool descriptions' describes the software tool ToolDog. ToolDog improves the interoperability of bio.tool-deposited entries within workbenches by converting their descriptions into formats that are compatible with workflow standards.

 ToolDog is a convenient addition to the existing capabilities for the integration of bio.tools entries with workbench environments.

 I found Figure 2 particularly interesting, describing the metadata coverage descriptions from two of the main Galaxy servers. Do you have the raw data with which this figure was created? It would be good to have it openly shared. Figure 2 illustrates the problem of the significant lack of completeness in crucial metadata descriptions of Galaxy tools.

 My main recommendation for this article would be to provide a step-by-step guide on how to run ToolDog using a self-contained example. I feel unable to test the tool because I do not know how to download the metadata from a bio.tools entry and need to set up my python environment, download the code and make it work. This article, although it is geared toward a programmer audience, it would be hard to test/reproduce for someone who is not a seasoned python programmer. I would thus recommend a beginner's guide for those of us who are not so technical.

 Other than that, I am glad to see all the source code adequately deposited both in github and Zenodo for the snapshot image for this publication. The MIT license is also commendable as it allows free reuse and modification.

 Finally some minor corrections:

- Link in the first paragraph of the results section ‘of a significant portion of the tool description’ is broken
- Link on the second paragraph of the results section ‘ https://github.com/khillion/galaxyxml-analysis/annotate_usegalaxy’ is broken
- Discussion section bullet point #3 ‘such web service’ ==> such as web services

I have read this submission. I believe that I have an appropriate level of expertise to confirm that it is of an acceptable scientific standard.

# Peer review: Referee response for version 1

**DOI:** 10.5256/f1000research.14069.r28565

**Published:** 2017-12-18

**Authors:** Christopher J. Fields

**Affiliations:** 1 High-Performance Biological Computing Group, Roy J. Carver Biotechnology Centre, University of Illinois at Urbana–Champaign, Urbana, IL, USA

The paper presents a very nice overview on how ToolDog is used to (1) generate new tool descriptors for Galaxy and CWL from code analysis, and (2) improve documentation for current tools from the bio.tools registry.  This provides a valuable service to the bioinformatics community and in particular to ensuring that tooling information is consistently described but also updatable. In my opinion this should be accepted, with some minor suggested revisions. 

 Speaking of ‘suggestions':

1. The current title ‘Using bio.tools to generate and annotate workbench tool descriptions’ suggests the paper will talk more generally about bio.tools, whereas the text focuses primarily on the specific component ToolDog. The title should be modified to reflect this.
2. The graphs in Fig.2 would be more effective if they were displayed in an integrated manner (single bar chart?), so that the improvements that ToolDog makes are more easily compared to one another.  
3. The discussion about the challenges in autogenerating tool documentation (language, code practices, etc), in the discussion, are spot-on. However not much is discussed on if / how ToolDog might address some of these challenges, though there are suggestions on how to more readily map existing tool descriptions to add to or update. Maybe this could be elaborated on, even if it’s indicating the problems may not be easily overcome? 
4. I’m wondering whether the information in Fig. 5 might be better displayed (or augmented) as a before / after comparison to more readily demonstrate how ToolDog could automatically improve tool descriptions. Another option is whether this information could be somehow connected to the data in Fig. 2 to show how ToolDog improves the overall documentation.

I have read this submission. I believe that I have an appropriate level of expertise to confirm that it is of an acceptable scientific standard.

# Peer review: Referee response for version 1

**DOI:** 10.5256/f1000research.14069.r28566

**Published:** 2017-12-15

**Authors:** Michael L. Heuer

**Affiliations:** 1 Department of Bioinformatics Research, National Marrow Donor Program (NMDP), Minneapolis, MN, USA; 2 AMPLab, University of California, Berkeley, CA, USA

From the point of view of a software tool author, it is not a simple task to provide high-quality metadata and software tool descriptions. Any tooling that supports DRY (don't repeat yourself) in this regard is most welcome.

 The authors describe a path from the bio.tools bioinformatics software registry, which uses a rich metadata schema for syntax, the EDAM ontology for semantics, and strongly written guidelines to ensure high-quality entries, to tool descriptions for the Galaxy workbench and in Common Workflow Language (CWL) for use on various workflow execution environments.

 Much of the metadata in the tool descriptions is generated by the ToolDog utility from an entry in bio.tools, ensuring proper mapping between metadata concepts. This would be a great help when bootstrapping Galaxy and CWL support for a new software tool. The authors also describe and implement a use case for enriching existing tool descriptions.

 I am curious if there are practical benefits to enriching tool descriptions with EDAM ontology terms, in addition to quality of metadata from using well defined terms from a controlled vocabulary?

 The source code is available at the Github link provided and is licensed MIT License as stated in the paper. I appreciate that the scripts and results of the analysis are archived as well.

I have read this submission. I believe that I have an appropriate level of expertise to confirm that it is of an acceptable scientific standard.
